# Case of Appendiceal Goblet Cell Adenocarcinoma With Gastric Metastasis

**DOI:** 10.14309/crj.0000000000002347

**Published:** 2026-10-21

**Authors:** A. Gautam, M. Jafferjee, K. Nimmagadda, M. Simien, M. Cimo

**Affiliations:** 1 Baylor Scott & White All Saints Medical Center, Fort Worth, TX; 2 Baylor Scott and White Digestive Diseases, Fort Worth, TX

**Keywords:** goblet cell adenocarcinoma, gastric metastasis, immunohistochemistry

## Abstract

Goblet cell adenocarcinoma (GCA) is a rare appendiceal neoplasm that typically metastasizes to the peritoneum and pelvic organs, with no reported gastric involvement. We report a 72-year-old man with multiple comorbidities presenting with recurrent small bowel obstruction. Imaging revealed a cecal and a gastric mass; colonoscopy confirmed poorly differentiated right-sided adenocarcinoma. Surgical resection with intraoperative biopsies identified high-grade appendiceal GCA infiltrating the cecum, mesentery, and gastric wall. Immunohistochemical staining showed cytokeratin 7 negativity and cytokeratin 20 positivity, suggesting an appendiceal origin. Endoscopic ultrasound-guided fine-needle aspiration of the gastric lesion confirmed gastric metastasis, demonstrating identical morphology and immunoprofile, thus expanding the metastatic spectrum of GCA.

## INTRODUCTION

Goblet cell adenocarcinoma (GCA) is a rare primary appendiceal adenocarcinoma, with an incidence of 0.05–0.3 per 100,000 individuals annually, according to North American registry studies. It accounts for approximately 14% of all primary appendiceal neoplasms.^1,2^ GCA is an aggressive tumor, with over 50% of cases showing metastasis at the time of diagnosis, predominantly to the peritoneum, pelvic organs, and omentum, and less commonly to the liver.^3–5^ We present a rare case of GCA of the appendix with metastasis to the stomach.

## CASE REPORT

This case involves a 72-year-old man with a history of type 2 diabetes mellitus, hypertension, coronary artery disease, kidney transplant on chronic immunosuppression, and diastolic heart failure who presented with recurrent small bowel obstruction.

One month ago, he had presented to another facility with intractable nausea, vomiting, abdominal pain, and distention. Cross-sectional imaging demonstrated obstruction at the ileocecal valve with associated lobulated thickening of the gastric wall. Conservative management resulted in symptomatic improvement, and he was discharged with plans for follow-up by gastroenterology due to concerns of a possible neoplasm.

He subsequently presented at our facility with similar complaints and had not followed up with gastroenterology in the interim. Relevant laboratory findings are summarized in Table 1. A computed tomography of the abdomen and pelvis revealed a distal small bowel obstruction extending to the level of the terminal ileum, with focal circumferential thickening of the cecal base near the ileocecal junction. A 4.9 × 3.7 × 8.1 cm hypodense mass was identified in the distal gastric body and antrum, concerning malignancy (Figure 1). Colonoscopy identified a right colonic mass, and biopsy confirmed poorly differentiated adenocarcinoma. Esophagogastroduodenoscopy (EGD) demonstrated a gastric lesion; however, biopsies were nondiagnostic, revealing only mild chronic gastritis.

**Table 1.** Laboratory results

Laboratory study	Results	Reference range
Sodium	138 mEq/L	136–145 mEq/L
Potassium	4.5 mEq/L	3.6–5.0 mEq/L
Chloride	109 mEq/L	98–107 mEq/L
Carbon dioxide	24 mEq/L	21–32 mEq/L
Creatinine	1.09 mg/dL	0.70–1.30 mg/dL
Carcinoembryonic antigen	<2.0 ng/mL	<2.5 ng/mL (nonsmoker) <5.0 ng/mL (smoker)
Cancer antigen 19.9	178 U/mL	<34 U/mL

**Figure 1.**
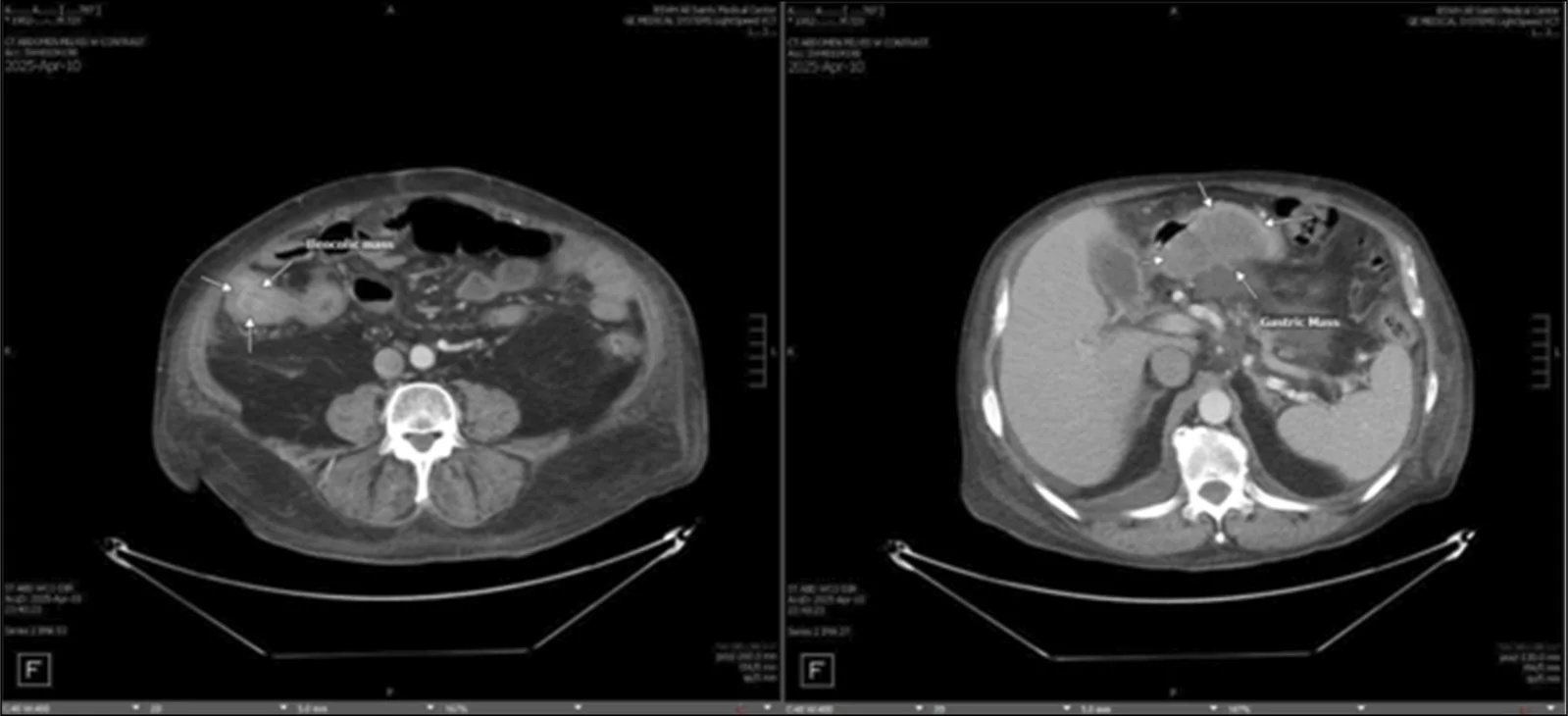
Computed tomography of the abdomen and pelvis with contrast showing ileocolic mass (left) and gastric mass (right).

Surgical resection with open right hemicolectomy was performed due to active bowel obstruction and the need for thorough exploration. Intraoperative gastric wall and mesenteric biopsies were also obtained in light of the concurrent gastric lesion in the radiography along with suspicious gastric wall.

Histopathology of the surgical specimen revealed high-grade appendiceal GCA with extension into the cecum, gastric wall, and mesentery (Figure 2). Six of nine lymph nodes were positive, and multiple tumor deposits were noted in peri-colonic adipose tissue, consistent with stage pT4bN2M1b disease. Immunohistochemistry (IHC) showed cytokeratin 20 (CK20), CDX2 positivity, cytokeratin 7 (CK7) negativity (Figure 3), patchy synaptophysin expression, rare chromogranin-positive cells, and a Ki-67 proliferation index of approximately 30%.

**Figure 2.**
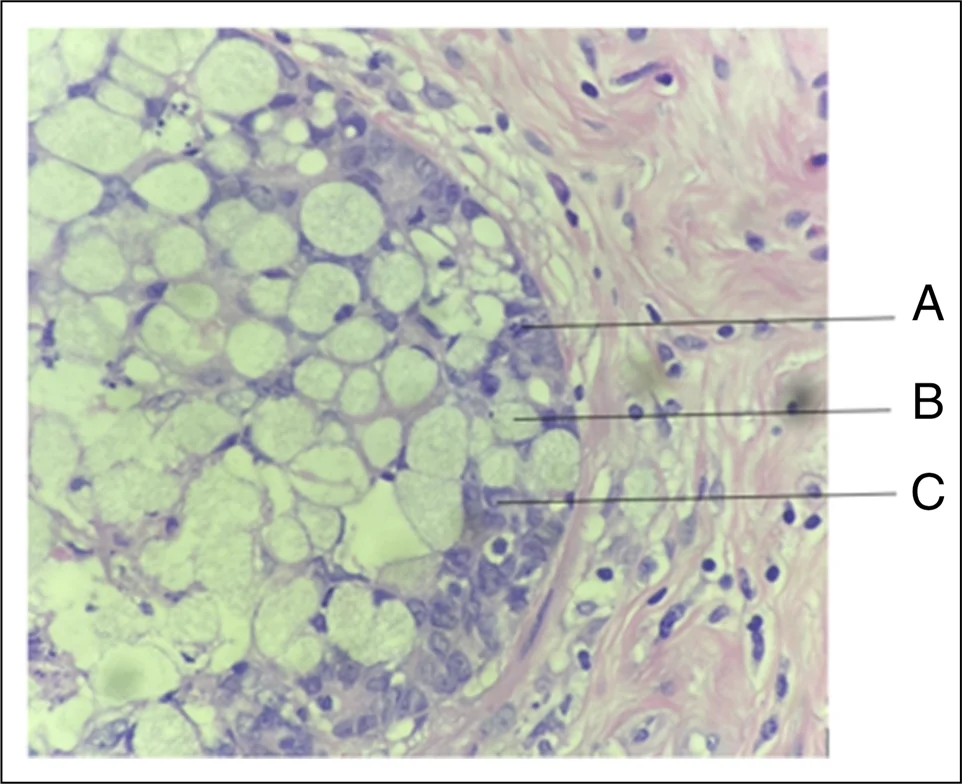
High-power (60×) hematoxylin and eosin stain image of infiltrating tumor with a single, rare mitotic figure (A); large mucin vacuoles (goblet cells) (B); and moderate nuclear pleomorphism (C).

**Figure 3.**
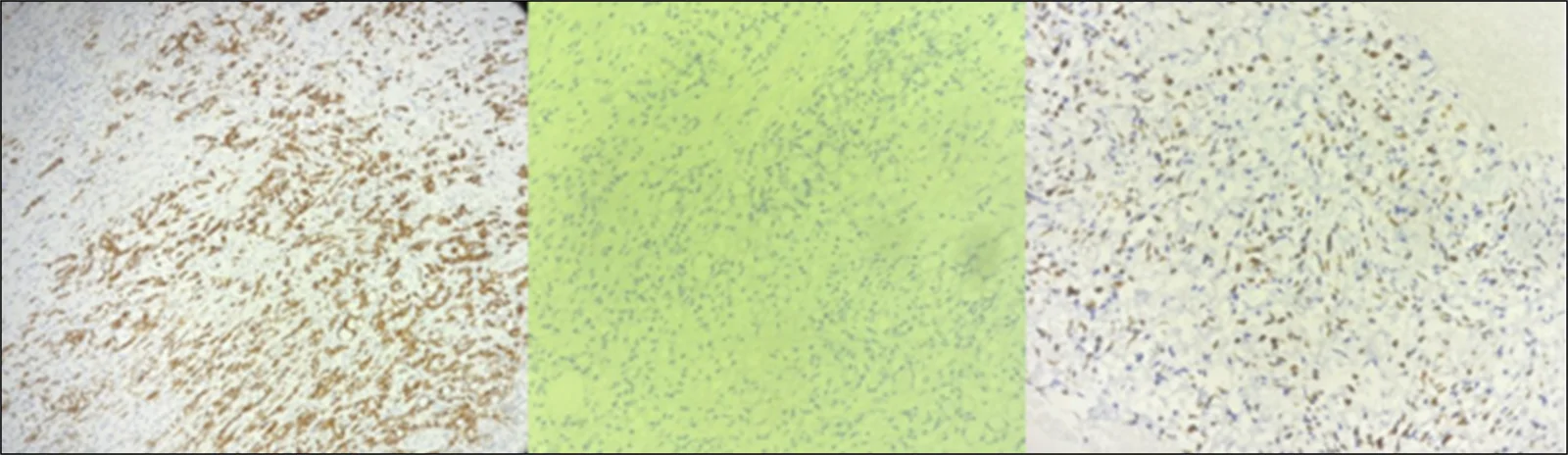
Immunohistochemical stain for CK20 positive (left) and CK7 negative (middle), highlighting the appendicular lesion, and CK20 positive (right), highlighting the gastric lesion. CK20, cytokeratin 20; CK7, cytokeratin 7.

While awaiting histopathology results, endoscopic ultrasound-guided fine-needle aspiration of the gastric mass was performed to obtain a more representative sample. This confirmed the presence of GCA, morphologically identical to the appendiceal primary, confirming metastatic involvement of the gastric wall (Figure 4). Palliative care was consulted, and after discussions with the patient and family, the decision was made to pursue hospice and comfort care.

**Figure 4.**
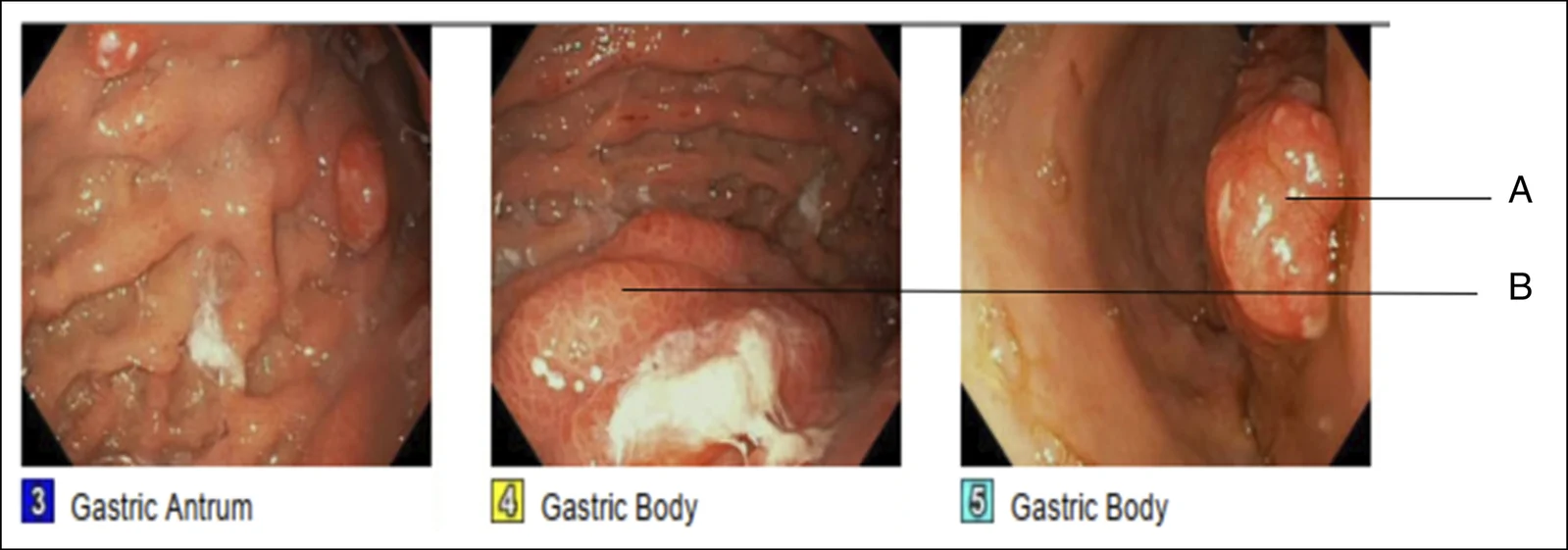
Endoscopy showed diffuse severe inflammation in the stomach (B) with polypoid lesion (A).

## DISCUSSION

GCA exhibits dual features of adenocarcinoma and neuroendocrine differentiation, characterized histologically by goblet-like mucinous cells (Figure 5) intermixed with variable numbers of endocrine and Paneth-like cells.^6^ It is exclusively classified as appendiceal tumors in the fifth edition of the WHO classification of digestive system tumors.^6^ While the appendix remains the prototypical site, isolated reports have noted GCA outside the appendix, including rare occurrences in the small bowel, colorectum, and stomach.^2,7,8^

**Figure 5.**
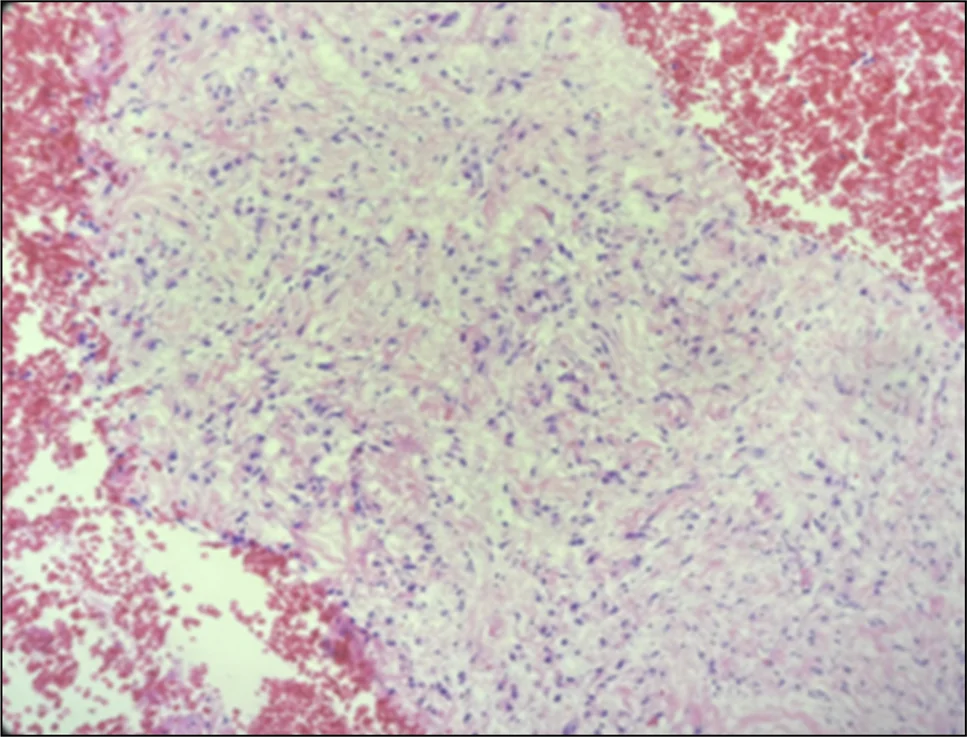
Low-power (10×) hematoxylin and eosin stain image of metastatic goblet cell adenocarcinoma of the appendix to the stomach.

The clinical presentation of GCA varies from vague abdominal pain in the early stages to an abdominal mass, bowel obstruction, or ascites in more advanced cases, as seen in our patient.^9^ GCA infiltrates diffusely^9^ and is rarely diagnosed endoscopically in early stages due to its submucosal growth.^10,11^ Histopathology, supported by IHC, remains the diagnostic gold standard, with right hemicolectomy being the standard treatment.^12–15^

GCA poses diagnostic challenges due to its histologic and clinical overlap with adenocarcinomas and neuroendocrine tumors making IHC essential. A CK7−/CK20+/CDX2 positive IHC pattern suggests appendiceal origin.^16^ In advanced cases, distinguishing metastasis from primary tumors requires careful morphological and clinical correlation with great emphasis on tumor distribution. GCA of the appendix tends to spread early to the peritoneum, especially to the ovaries and regional lymph nodes,^9^ with rare hematogenous metastases to the liver and lung in advanced disease.^17,18^

In this case, the significant masses in both the stomach and appendix posed a challenge to establish the primary origin of tumor in our patient. Correct diagnostic classification is adamant to guide management. Several key factors, however, supported the appendix as the primary site.

Although CK7 negativity and CK20/CDX2 positivity are more consistent with appendix origin, these immunohistochemical results alone are not definitive. Several additional key factors, when taken together, help make the diagnosis of an appendix primary much more robust (Figure 6).

**Figure 6.**
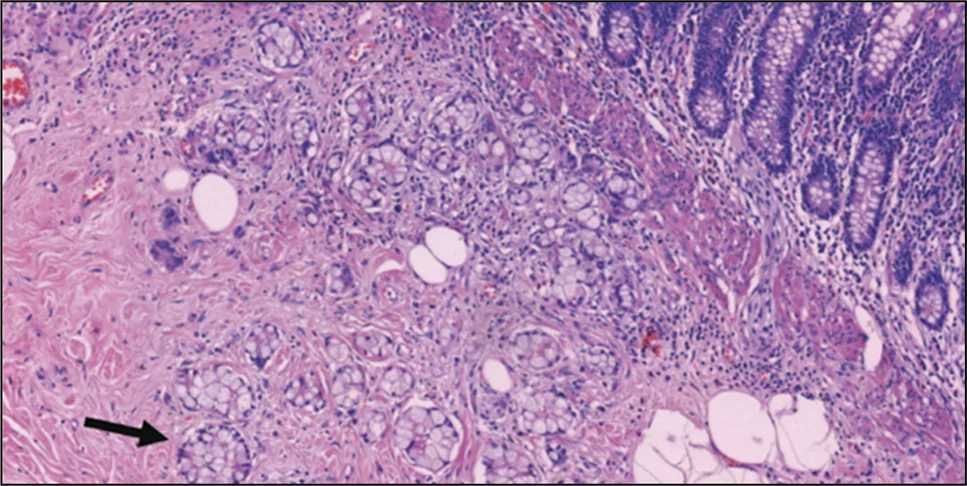
Low-grade goblet cell adenocarcinoma of the appendix showing clusters of tumor cells with goblet-like mucinous cells, (arrow) Cluster of goblet cells. Image Reproduced from Karaoğlan et al. Turk J Colorectal Dis. 2024;34(2):66–68, under CC BY license.

First, the appendix is the most common origin of GCA, with goblet cells normally found in the intestinal tract but not in the gastric mucosa.^19^ Second, there are only a few cases of primary gastric GCA that have been described in the literature, and the diagnosis of primary extra-appendiceal GCA should only be made after excluding an appendiceal origin.^20^ Last, pathological examination revealed a higher density of tumor cells within the appendix compared with the gastric mass, with extensive regional metastasis. IHC further supported an appendiceal origin, with CK7 negativity and CK20 positivity in both the appendiceal and gastric lesions. We hypothesize that peritoneal spread is the most likely route of metastasis in this case, given the extensive regional lymph node and mesenteric involvement; however, direct spread cannot be ruled out given extensive spread. In addition, composite assessment of morphology, immunophenotype, anatomic context, and clinical pattern of spread distinguishes it from colorectal cancer.

This case expands the recognized metastatic profile of appendiceal GCA. It highlights the importance of maintaining a broad differential when evaluating gastric masses in patients with known GCA. It also underscores the critical need for thorough intraoperative assessment and biopsy of any suspicious gastric lesions. Failure to consider metastatic disease may lead to diagnostic misclassification and affect management. As this was a metastatic lesion, the patient underwent right hemicolectomy, whereas primary gastric GCA would be managed by neoadjuvant chemotherapy and gastrectomy based on the extent of spread. By documenting gastric metastasis from appendiceal GCA, this report contributes to the evolving understanding of this rare malignancy and highlights the pivotal role of histopathology and IHC in distinguishing primary gastric tumors from metastatic involvement.

This report presents the first documented case of appendiceal GCA metastasizing to the stomach, illustrating the diagnostic complexities associated with such rare metastatic spread. Accurate differentiation between primary and metastatic lesions in cases like this necessitates a comprehensive diagnostic approach, including imaging, endoscopic evaluation, and biopsies—both preoperative and intraoperative—of the gastrointestinal lesions in question, along with immunohistochemical analysis. The findings underscore the importance of considering metastatic disease in the differential diagnosis of gastric masses, particularly in patients with a history of appendiceal GCA.

## DISCLOSURES

Author contributions: A. Gautam: literature review, original manuscript writing, conceptualization, collecting pictures, coordination with team, and manuscript editing. M. Jafferjee and K. Nimmagadda: original manuscript writing and editing. M. Simien: conceptualization, mentorship, and manuscript editing. M. Cimo: evaluated and described the pathology slides. A. Gautam is the article guarantor.

Financial disclosure: None to report.

Informed consent was obtained for this case report.

## ABBREVIATIONS:

CK20: cytokeratin 20
CK7: cytokeratin 7
EGD: esophagogastroduodenoscopy
GCA: goblet cell adenocarcinoma
IHC: immunohistochemistry
